# Cryopreservation of African painted dog (*Lycaon pictus*) ovarian tissue

**DOI:** 10.3389/fvets.2023.1134726

**Published:** 2023-03-17

**Authors:** Kate E. Hartzler, Chiara McCartney, Nucharin Songsasen, Jennifer B. Nagashima

**Affiliations:** Center for Species Survival, Smithsonian National Zoo and Conservation Biology Institute, Front Royal, VA, United States

**Keywords:** canid, vitrification and thawing, ovarian tissue, slow freezing cryopreservation, African painted dog

## Abstract

Development of techniques for the preservation and use of gonadal tissues are increasingly needed for the genetic management of the endangered African painted dog (*Lycaon pictus*). Here we evaluated two cryopreservation techniques for ovarian tissue (2 × 2 × 1 mm^3^ fragments, *n* = 11 individuals): needle immersed vitrification (NIV), with equilibration in a 7.5% dimethyl sulfoxide (DMSO) and 7.5% ethylene glycol (EG) solution, and vitrification in a 15% DMSO, 15% EG, and 0.5 M sucrose solution, and slow freezing in cryovials with either the equilibration (SF-E) or vitrification (SF-V) solutions. Following warming, tissues were either fixed and embedded for evaluation of density of morphologically normal follicles, semi-quantitative scoring of stromal cell preservation, and apoptotic index (TUNEL stain), and/or flash-frozen for expression of proliferation (*PCNA*), apoptosis (*CASP3, BCL2*), or oxidative stress (*GPX3, SOD1, SOD2*) pathway genes (*n* = 4). Needle immersed vitrification maintained higher density of morphologically normal follicles compared to the slow freezing protocols applied (*p* < 0.05), with no significant changes in expression of select genes among treatment groups. A slight increase in apoptotic index was observed in all cryopreservation groups, but only reached significance in SF-E compared with fresh tissue controls (*p* < 0.05). Future research should be dedicated to developing improved methods for ovarian tissue culture in the species, both as a means to evaluate the efficacy of tissue cryopreservation techniques and for the production of viable oocytes from banked ovarian tissue in the endangered African painted dog.

## Introduction

The population of the endangered African painted dog, also known as the African wild dog or cape hunting dog (*Lycaon pictus*), has shown continued decline over the past 30 years. There are estimated to be fewer than 7,000 individuals remaining in the wild, due primarily to habitat fragmentation, human-wildlife conflict, and spread of infectious diseases from domestic dogs (1). As a consequence, genetic management of the *ex situ* population has become increasingly important for species survival. Though in early stages of development for canid species, there is mounting interest in the optimization and application of various assisted reproductive technologies (ART) toward this goal. For example, in the event of individual death or lack of natural breeding, the ability to collect and cryopreserve gonadal tissue would allow for the long-term banking of germplasms that could be used to re-infuse critical genetics into the population in the future. These technologies would be invaluable for the sustainability of a genetically viable population of African painted dogs.

Ovarian tissue cryopreservation (OTC) is increasingly being used in human medicine as a method of preserving fertility for women (2). As of 2019, over 100 pregnancies and nearly 60 human births have been achieved through OTC and auto-transplantation, according to one meta-analysis (3). OTC is also being applied to various animal species to preserve genetic material until such a time that *in vitro* fertilization or allo- or xeno-grafting can be performed. Thus far, transplantation of cryopreserved ovarian tissue has been reported to result in live birth in mice and sheep (4, 5), and the return of fertility in cats (6). Xenografting of ovarian tissues into immunocompromised mice has allowed for the successful growth of primordial and antral follicles across a number of species, including the African elephant (*Loxodonta africana*) (7), common wombat (*Vombatus ursinus*) (8), and lion (*Panthera leo*) (9). These examples show that OTC and post-thaw tissue use is gaining momentum; however, the lack of births in non-human species indicate there is much research yet to be done. Widespread incorporation of OTC into endangered species management plans has been particularly limited, due to the lack of species-specific protocols for the majority of our wildlife animals.

In most species in which OTC has been studied, two primary methods of cryopreservation have been used—slow freezing and vitrification. Slow freezing (SF) is the process of a controlled, gradual decrease in temperature in the presence of cryoprotective agents (CPAs) to protect against ice formation and subsequent cell damage, before long-term tissue storage in liquid nitrogen. Conversely, tissues are typically exposed to elevated concentrations of CPAs for a shorter duration preceding ultrarapid cooling to achieve vitrification, minimizing or eliminating ice crystal formation in the sample. One version of this method previously applied to ovarian tissue specifically is needle immersed vitrification (NIV) (10), wherein tissues are loaded onto small-gauge needles during equilibration with CPAs, then plunged directly into liquid nitrogen. In human tissues, NIV (with 13.5% DMSO and EG, and 0.5 M sucrose) allowed for the reduction in CPA concentrations compared with dropping vitrification (20% DMSO and EG), while decreasing apoptosis (11). In both human and murine ovarian tissue, NIV has been shown to better support morphologically normal follicle proportions and stromal cell architecture compared with slow freezing (10); however, slow freezing was advantageous to NIV for the maintenance of testicular tissue architecture in the gray wolf (12). Both slow freezing and vitrification have been evaluated for domestic dog (*Canis familiaris*) ovarian tissue (13), and a number of species have demonstrated tissue viability following autografting of cryopreserved ovarian tissue [reviewed in (14)].

To date, there have been no studies focused on cryopreservation of African painted dog ovarian tissues. This study aims to compare the effects of needle immersed vitrification vs. slow freezing on ovarian stromal cell architecture, follicle density, tissue viability, and gene expression, toward the goal of developing a successful cryopreservation protocol for genome preservation in the African painted dog.

## Materials and methods

### Chemicals

Unless otherwise indicated, all chemicals were purchased from Sigma-Aldrich Chemical Company (St. Louis, MO).

### Animals

Ovarian tissues were obtained opportunistically from 11 African painted dogs (aged 2–6 years) from Association of Zoos and Aquarium's Species Survival Program institutions undergoing routine ovariohysterectomy for population management purposes or during necropsy between 2017 and 2021. These included three sets of littermates. Donated tissues were shipped overnight on ice to the laboratory at Smithsonian National Zoo and Conservation Biology Institute (NZCBI, Front Royal, VA) in saline-soaked gauze.

### Tissue processing and cryopreservation

**Figure 1:**
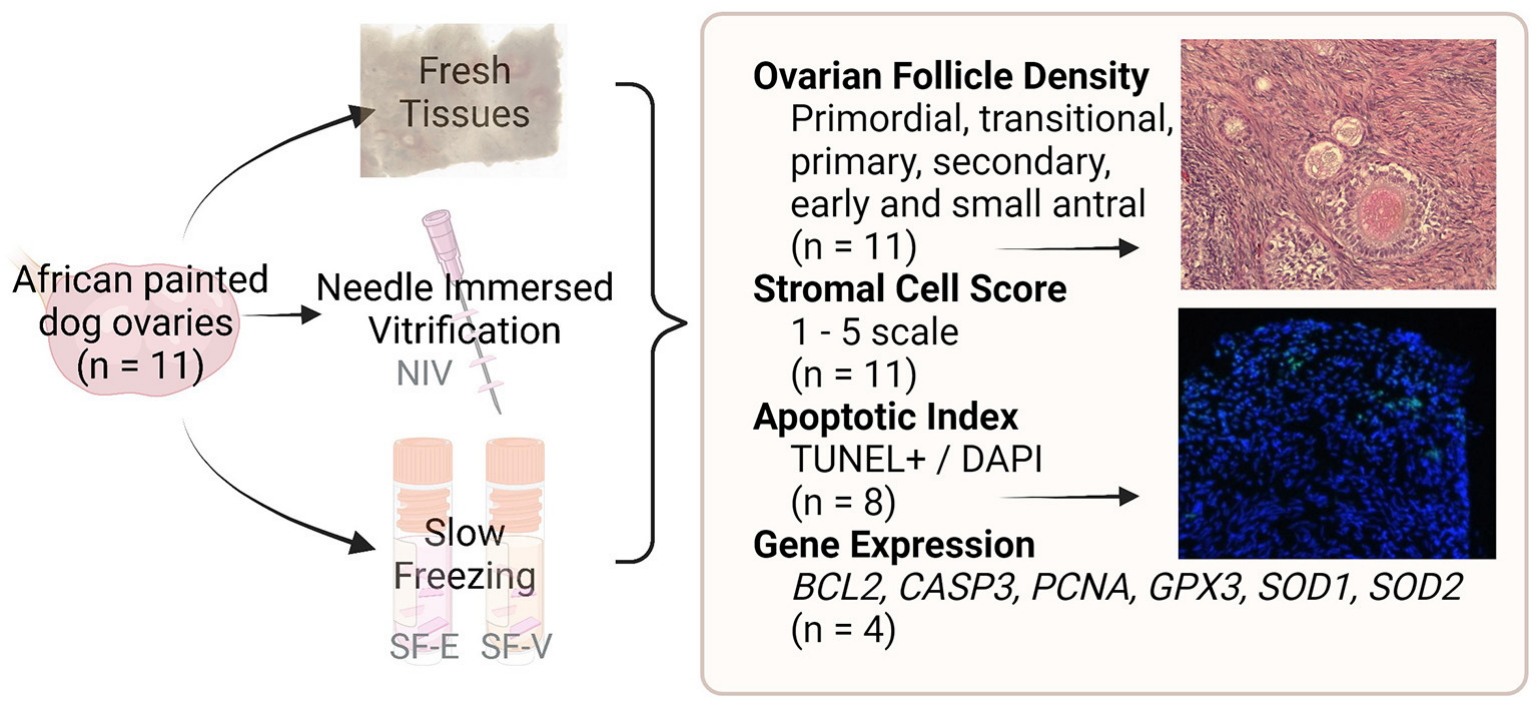
Study design flowchart. African painted dog ovaries (*n* = 11 individuals) were processed for either needle immersed vitrification (NIV) or slow freezing with either 7.5% DMSO and EG (SF-E), or 15% DMSO and EG with 0.5M sucrose (SF-V). Fresh tissues, as well as post-warming cryopreserved tissues were fixed or flash-frozen for evaluation of ovarian follicle density (*n* = 11), stromal cell architecture (*n* = 11), apoptosis (*n* = 8), or gene expression (*n* = 4).

Ovaries were transferred to handling medium consisting of Minimum Essential Medium Alpha modification supplemented with 0.1 g/ml BSA, 100 U/ml penicillin g sodium salt, 100 μg/ml streptomycin sulfate, and 10 mM HEPES (12). Ovarian dimensions (length, width, height) were measured and used to calculate ovarian volume for each individual. The ovaries were then dissected into small (~2 × 2 × 1 mm^3^) fragments. Four to five fragments of fresh tissue per donor were fixed overnight in 4% paraformaldehyde and then transferred to 70% ethanol until histological processing. Additional fragments were flash frozen in liquid nitrogen for gene expression analyses as Fresh controls. The remaining tissue pieces were either preserved using a modified slow freezing method (15) or using the needle immersion vitrification (NIV) protocol adapted from Mouttham and Comizzoli (16) (detailed below). Following warming, tissues from each treatment group and individual were either fixed for histological evaluations or flash-frozen for subsequent gene expression analyses (study design flowchart, Figure 1).

### Needle immersed vitrification and warming

Needle immersed vitrification (NIV) was performed using an adapted protocol previously optimized for feline ovarian tissue vitrification (16). Four to five ovarian tissue segments were threaded onto a 30 G needle (BD PrecisionGlide needle, Fisher Scientific) with space between each fragment. The needles were transferred to an equilibration solution consisting of 20% FBS in MEM alpha with 7.5% EG and 7.5% DMSO for 10 min at 4°C, then transferred to a vitrification solution consisting of MEM with 20% FBS supplemented with 15% EG, 15% DMSO, and 0.5 M sucrose for 10 min at 4°C. The tissue-laden needles were then removed from the vitrification solution, excess solution was quickly absorbed using Kimwipe (KimTech), and the needles were directly plunged into liquid nitrogen. Needles were then transferred to a cryovial and stored in liquid nitrogen. Warming was performed by removing the needles from the cryovials in liquid nitrogen and quickly transferring them sequentially through warmed (37°C) MEM alpha with 20% FBS supplemented with 1 M, 0.5 M, and 0.25 M sucrose for 5 min each. Following warming, two fragments/freezing condition/animal were fixed in 4% paraformaldehyde solution in PBS for histological evaluations, and one to two fragments/condition/animal were flash-frozen in liquid nitrogen for gene expression analyses.

### Slow freezing and thawing

Slow freezing medium consisted of Minimum Essential Medium Alpha modification (MEM) with 20% FBS and either 7.5% DMSO and 7.5% EG (SF-E) or 15% DMSO, 15% EG, and 0.5 M sucrose (SF-V). Four to five ovarian sections were immersed in 0.5 ml of each freezing medium in a 2.0 ml cryovial (Fisher Scientific, Pittsburg, PA). The cryovials were then placed into a Nalgene freezing container (Mr. Frosty, Fisher Scientific) and transferred into a −80°C freezer overnight to provide a cooling rate of ~ −1°C/min. Cryovials were subsequently plunged into a liquid nitrogen and stored until evaluation. Warming was performed by removing the cryovials from liquid nitrogen and then immersing them in a 37°C water bath for 1 min. One milliliter of warmed (37°C) MEM with 1 M sucrose was slowly added to each vial and the contents transferred to a sterile Petri dish with 1 M sucrose solution. After 5 min, the tissues were transferred sequentially to Petri dishes containing MEM with 0.5 M and 0.25 M sucrose for 5 min each. Two segments/freezing condition/donor were fixed in 4% paraformaldehyde solution, and one to two fragments/condition/animal were flash-frozen in liquid nitrogen for gene expression analyses.

### Histological tissue processing and evaluations

Fixed tissues (*n* = 11 individuals) were dehydrated *via* an ethanol gradient prior to embedding in Paraplast^®^ paraffin blocks. Sections (6 μm thick) were cut using a microtome (ReichertJung Leica 2030 Biocut), and every 10th section was mounted on a slide and processed for histological evaluation. For evaluation, five sections were mounted per slide, representing ~300 μm depth into the tissue. Hematoxylin and eosin-stained sections were imaged using an EVOS autoFL2 microscope (Thermo-Fisher) under 40× magnification. Stromal preservation was evaluated semi-quantitatively. The stromal tissue of each section of tissue was scored from 1 to 5, with a score of 1 indicating the stromal tissue was entirely damaged (pyknotic or absent stromal cell nuclei, and/or with irregular patterning or orientation). Scores of 2, 3 and 4 marked tissues with ~75%, 50%, or 25% damaged stroma, respectively, whereas a score of 5 indicated the stromal tissue of the entire section was viable and retained normal architecture. The scores for the two fixed tissue sections per individual were averaged to determine the score for that animal in that treatment.

Assessment of ovarian tissue area and morphologically normal follicle counts were performed with follicles staged as primordial, transitional, primary, secondary, early antral, and antral (17). Each follicle type and the total number of follicles per tissue was divided by the tissue area to determine density of morphologically normal follicles per individual per treatment. Normal follicles were those that retained structure and had a nucleus with active chromatin vs. follicles with pyknotic nuclei and loss of structure.

### Gene expression

RNA extraction was performed on flash-frozen fresh and post-thawed tissue samples (*n* = 4 individuals) using the Qiagen RNeasy Plus Kit, per manufacturer's instructions, followed by cDNA preparation using the Invitrogen SuperScript™ III First-Strand Synthesis System with 50 ng/ul RNA per reaction. As Nanodrop One (ThermoFisher Scientific) quantification indicated carry-over chemical presence in extracted RNA, the PureLink PCR Purification kit (Invitrogen) was used to further purify subsequent cDNA products. RT-PCR was performed using the Roche FastStart Essential DNA Green Master kit and run on the Roche LightCycler 96. Due to low amounts of high quality and quantity purified RNA, only 4 individuals were able to be assessed for all treatment groups and genes, as other individuals yielded incomplete sets of data.

Beta actin was utilized as the housekeeping gene (18). For apoptosis and proliferation, caspase 3 (*CASP3*), B cell lymphoma 2 (*BCL2*), and proliferating cell nuclear antigen (*PCNA*), were evaluated. BCL2 is part of the mitochondrial anti-apoptotic pathway (19), and has been shown to be elevated in fresh vs. vitrified and slow frozen human ovarian tissue (20). Oxidative stress response pathway members, including glutathione peroxidase 3 (*GPX3*) and superoxide dismutase 1 and 2 (*SOD1, SOD2*) were also assessed. GPX3 protects cells from oxidative damage *via* reducing hydrogen peroxide, and has been indicated to be elevated in DMSO-exposed murine zygotes even prior to cryopreservation (21).

Primers were designed as previously described (18) to align to areas of high homology between at least three of the following carnivora: domestic cat (C), dog (D), ferret (F), and giant panda (P) mRNA transcripts (from the NCBI database https://www.ncbi.nlm.nih.gov/nucleotide/) for *GPX3, BLC2, SOD1, SOD2, and PCNA*. Alignments were performed using the Ensembl online tool (https://www.ensembl.org/), and primers were selected in NCBI Primer-BLAST (https://www.ncbi.nlm.nih.gov/tools/primer-blast/) (Table 1). Primer efficiency was calculated based on the slope of a standard curve, and variability accounted for in relative gene expression analyses *via* Pfaffl method (22).

**Table 1:** RT-PCR primer details.

**Name**	**Sequence**	**Size (bp)**	**Efficiency**	**Accession numbers**
*ACTB*	F: CCAACTGGGACGACATGGAGAAGATC R: CTCCGTGAGGATCTTCATGAGGTAGT	151	128%	C: XM_006941899
				D: ENSCAFT00000025413
				F: ENSMPUT00000016755
				P: ENSAMET00000007257
*BCL2*	F: CATGCCAAGAGGGAAACACCAGA R: GTGCTTTGCATTCTTGGATGAGGG	76	104%	C: NM_001009340.1
				D: NM_001002949.1
				F: XM_045065700.1
				P: XM_034642537.1
*GPX3*	F: GGAAGAGCTTGCGCCATTTG R: CTCCTGGCTCCTGTTTTCCG	74	89%	C: XM_003981387.6
				D: NM_001164454.1
				F: XM_004737800.3
				P: XM_002920757.4
*PCNA*	F: TCTGCAAGTGGAGAACTAGGA R: GTTACTGTAGGAGAGAGCGGA	170	92%	C: XM_003983740.5
				D: XM_038433208.1
				F: XM_004751519.3
				P: XM_002924824.4
*CASP3*	F: TTGCGTGCTTCTAAGCCAT R: TCTACAGTAATCTCCTCGGAAG	103	147%	C: NM_001009338.1
				D: XM_038690235.1
				F: EU836038.1
				P: XM_002914016.4
*SOD1*	F: AGGGCACCATCCACTTCGT R: GTCAGCCCTGTAATGGTTCC	70	113%	C: MH882489.1
				D: NM_001003035.1
				F: XM_013058840.2
				P: XM_002928799.4
*SOD2*	F: AGCCTGGGCTCAAGTTCAAT R: GGACACCGACGGATACAGTG	174	149%	C: XM_023254547.2
				D: XM_038654563.1
				P: XM_034669402.1

Described or predicted mRNA sequence accession numbers for carnivora members domestic cat [*Felis catus*, (C)], domestic dog [*Canis lupus familiaris*, (D)], ferret [*Mustela putorius furo*, (F)], and giant panda [*Aliuropoda melanoleuca*, (P)] are listed.

### Terminal deoxynucleotidyl transferase dUTP nick end labeling (TUNEL) staining

The ApopTag Fluorescein *In Situ* Apoptosis Detection Kit (Millipore Sigma), a TUNEL assay, was used per manufacturer instructions, with 0.5 ug/ml DAPI (4′,6-diamidino-2-phenylindole) counterstaining (*n* = 8 animals). One section per slide was treated with DNase for 15 mins following dewaxing as a positive control. Apoptotic fluorescent signal relative to DAPI per section was quantified in ImageJ as previously described (12), and reported as “apoptotic index.”

### Statistical analyses

Data presented as mean ± standard deviation. Data were analyzed in R Studio (version 4.1.0). Morphologically normal follicle density (per stage), stromal cell score, and apoptotic index were analyzed by Linear Mixed-effects Model (LME) with Tukey HSD *post-hoc* test using package lme4 (ver. 1.1.27) (23), following data transformation to achieve normality, where treatment (fresh, NIV, SF-E, and SF-V) was a fixed factor, and individual African painted dogs were the random effect. Differences in gene expression among treatment groups were analyzed *via* non-parametric Kruskal-Wallis test with Bonferroni correction.

## Results

**Figure 2:**
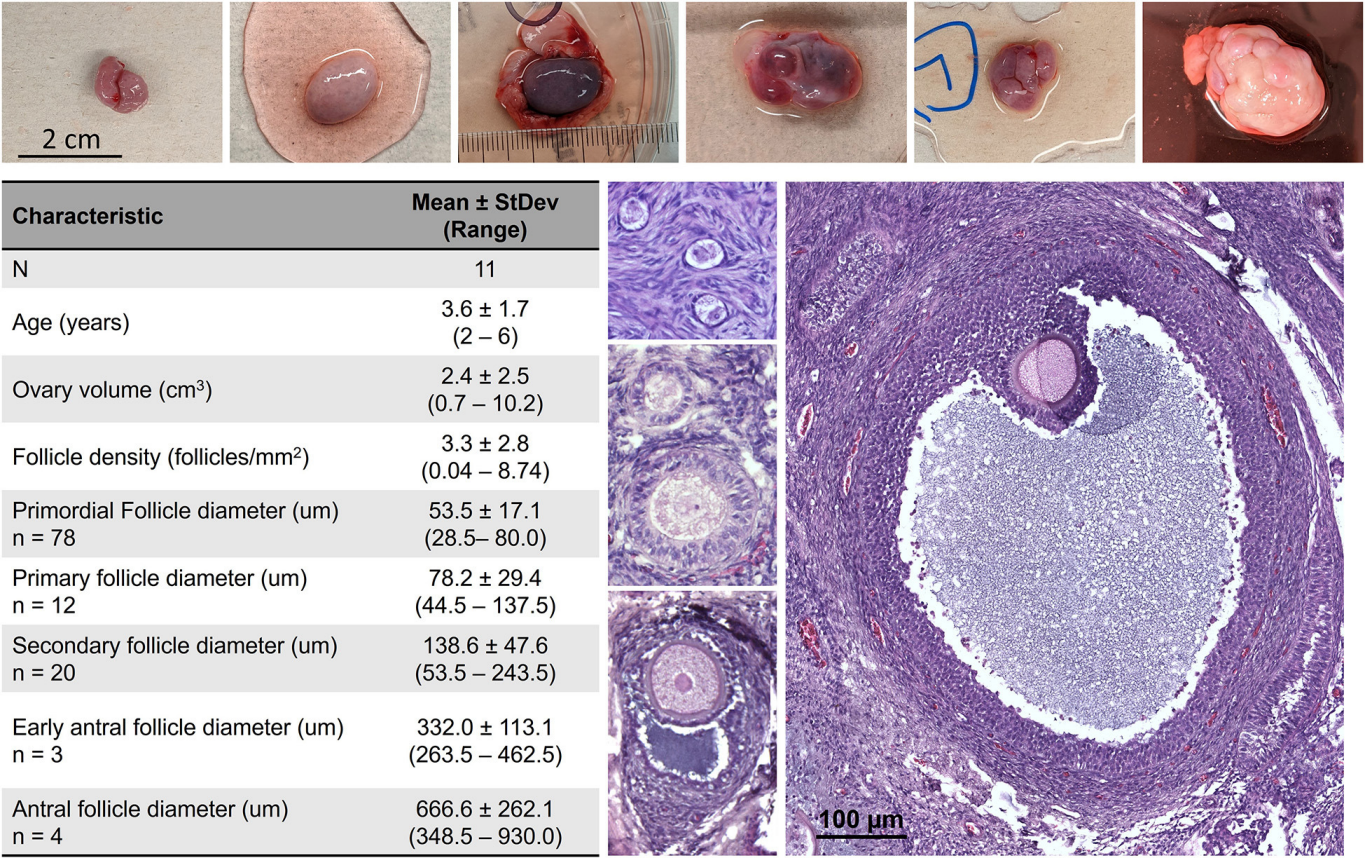
African painted dog (*n* = 11) ovarian tissue characteristics, with representative photos of ovaries and histological images of primordial, transitional, primary, secondary, early antral, and antral stage follicles. Antral stage follicles sizes reported were limited by the thickness of cortical tissue (1 mm) preserved for analysis.

As there is yet limited information on the morphometrics of the average African painted dog ovary and follicles, these were evaluated in fresh tissues (Figure 2). All individuals assessed (*n* = 11) represented females in peak reproductive years, with either themselves or their littermates having previously produced offspring. Ovarian tissues were obtained *via* ovariohysterectomy due to management needs rather than reproductive abnormality, and therefore represent a subset of the normal, healthy population. However, variation in ovarian volume as well as stage of the reproductive cycle (with large antral follicles and/or corpora lutea often accounting for the larger ovarian dimensions) was noted among individuals, and antral stage follicles sizes reported were limited by the thickness of cortical tissue (1 mm) preserved for analysis. Fresh African painted dog primordial, primary, and secondary stage follicles were, on average, up to 2× larger in the African painted dog compared with domestic dogs. For example, domestic dog primordial follicles average ~25 μm in diameter (13, 24), whereas for secondary follicles, domestic dog reports have ranged from 101.6 μm (13) to 131.2 μm (24) on average, which overlaps with the lower end of the sizes observed in African painted dog ovaries here.

**Figure 3:**
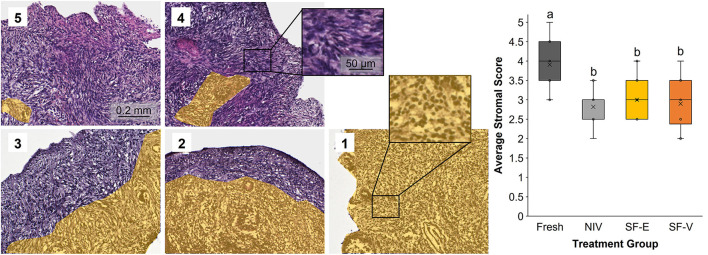
Stromal cell integrity in Fresh vs. post-thawed African painted dog ovarian tissues cryopreserved with needle immersed vitrification (NIV), or slow freezing with either 7.5% DMSO and EG (SF-E), or 15% DMSO and EG with 0.5 M sucrose (SF-V), with representative images of the scoring system **(1-5)** containing yellow overlay identifying areas with contiguous stromal cell disruption or atresia. Box-and-whisker plot display median, quartile, and data range, with letters indicating statistically significant differences among treatment groups following linear mixed model and *post hoc* Tukey's HSD test (*p* < 0.05).

**Figure 4:**
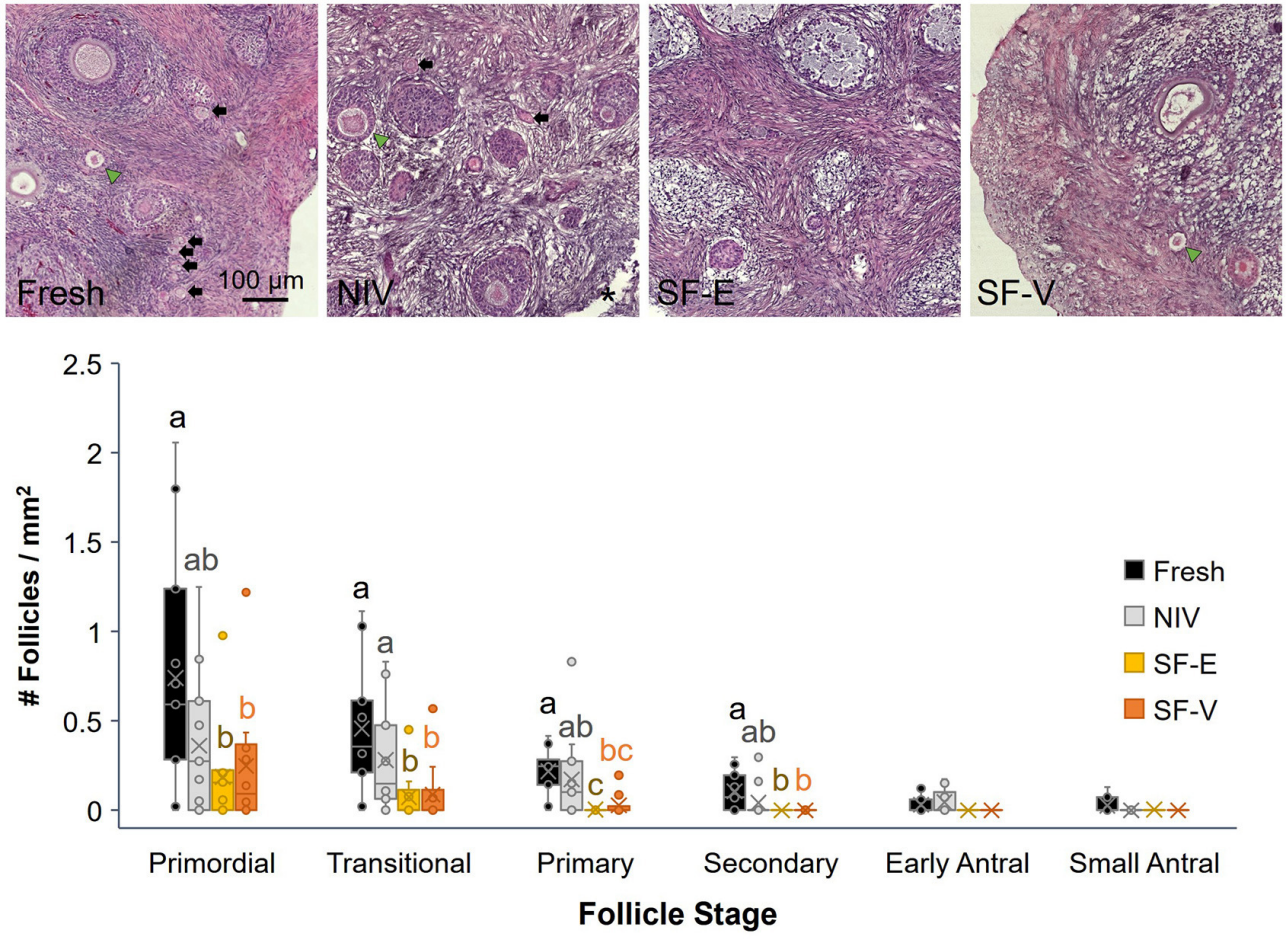
Density (number/mm^2^ tissue) of primordial, transitional, primary, secondary, early and small antral stage follicles in Fresh or cryopreserved-warmed [*via* needle immersed vitrification (NIV), or slow-freezing with either 7.5% DMSO and EG (SF-E), or 15% DMSO and EG with 0.5 M sucrose (SF-V)] African painted dog ovarian tissues (*n* = 11), with representative images from a single individual (top) where morphologically normal follicles (black arrows), abnormal follicles (green arrow heads), and needle penetration site (asterisk *) are indicated. Box-and-whisker plots display median, quartile, and data range for follicle densities, with circles representing individuals. Letters indicate statistically significant differences in density among treatment groups per follicle stage following linear mixed model and *post-hoc* Tukey HSD test (*p* < 0.05).

Semi-quantitative evaluation of stromal integrity revealed a significant (*p* < 0.01) difference between fresh tissue and all cryopreserved treatment groups (Figure 3). However, there was no difference in stromal integrity among the cryopreservation strategies. Comparatively, there was a statistically significant decrease in viable primordial follicle density between the fresh tissue and both the SF-E and SF-V groups (*p* < 0.05), with no differences between NIV and Fresh (Figure 4). The differences between the slow frozen treatment groups were also significant for transitional stage follicles compared with fresh (*p* < 0.001), as well as NIV tissues (*p* < 0.05). Primary follicles followed a similar trend for SF-E compared to NIV (*p* < 0.01) and SF-E and SF-V relative to fresh tissue (*p* < 0.001). No significant differences were identified for secondary, early antral, or antral follicle densities among treatment groups; however, evidence of atretic follicles of these advanced stages were observed in the post-thaw groups (Figure 4).

**Figure 5:**
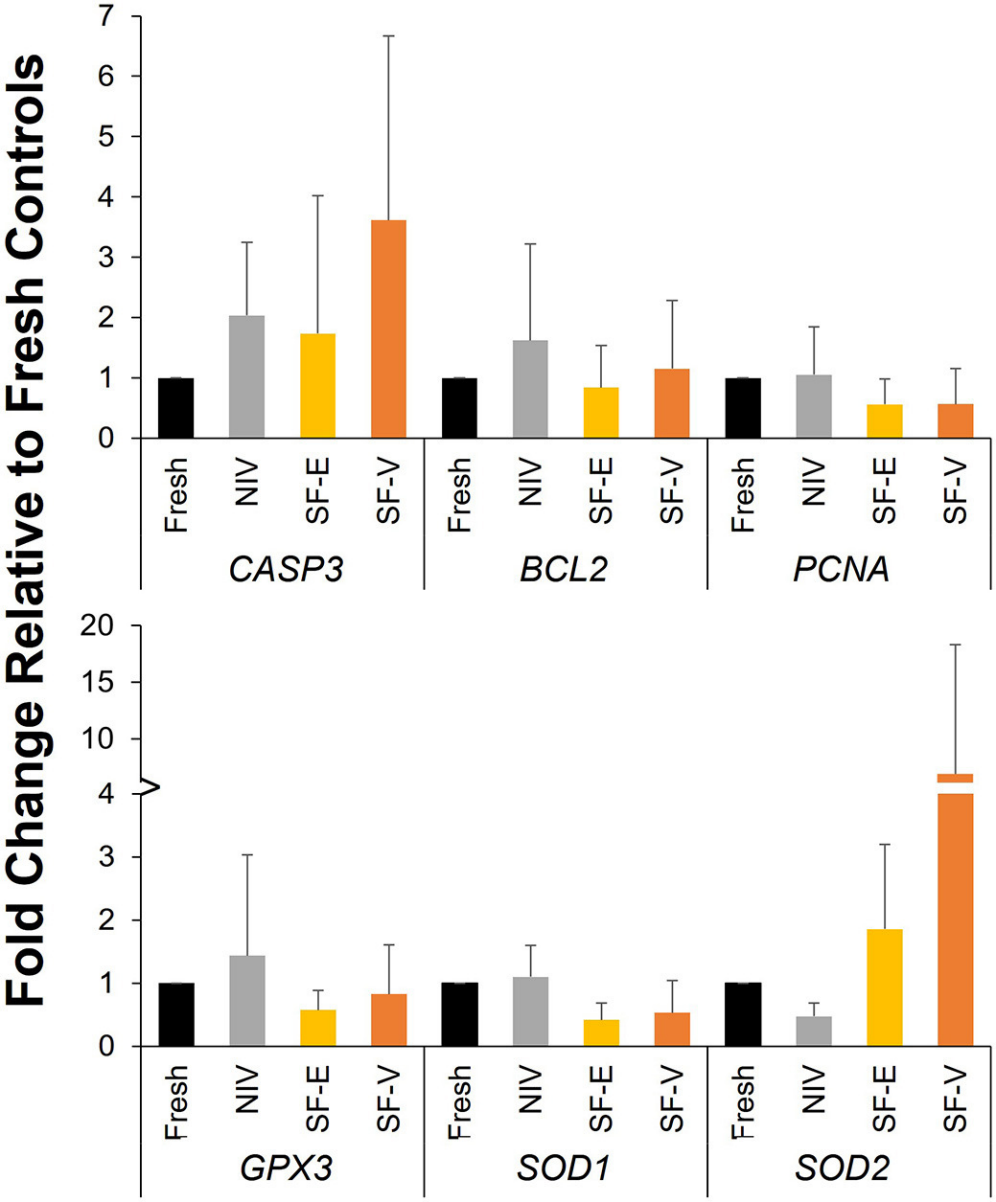
Gene expression in cryopreserved-warmed [*via* needle immersed vitrification (NIV), or slow freezing with either 7.5% DMSO and EG (SF-E), or 15% DMSO and EG with 0.5 M sucrose (SF-V)] African painted dog ovarian tissue relative to Fresh controls (*n* = 4). Y axis represents the fold change (mean ± standard deviation) in gene expression relative to Fresh controls.

Analysis of gene expression data (Figure 5) revealed no statistically significant differences among treatment groups, though somewhat higher levels of *CASP3* were observed in all cryopreservation treatments relative to fresh tissue (*p* > 0.05).

**Figure 6:**
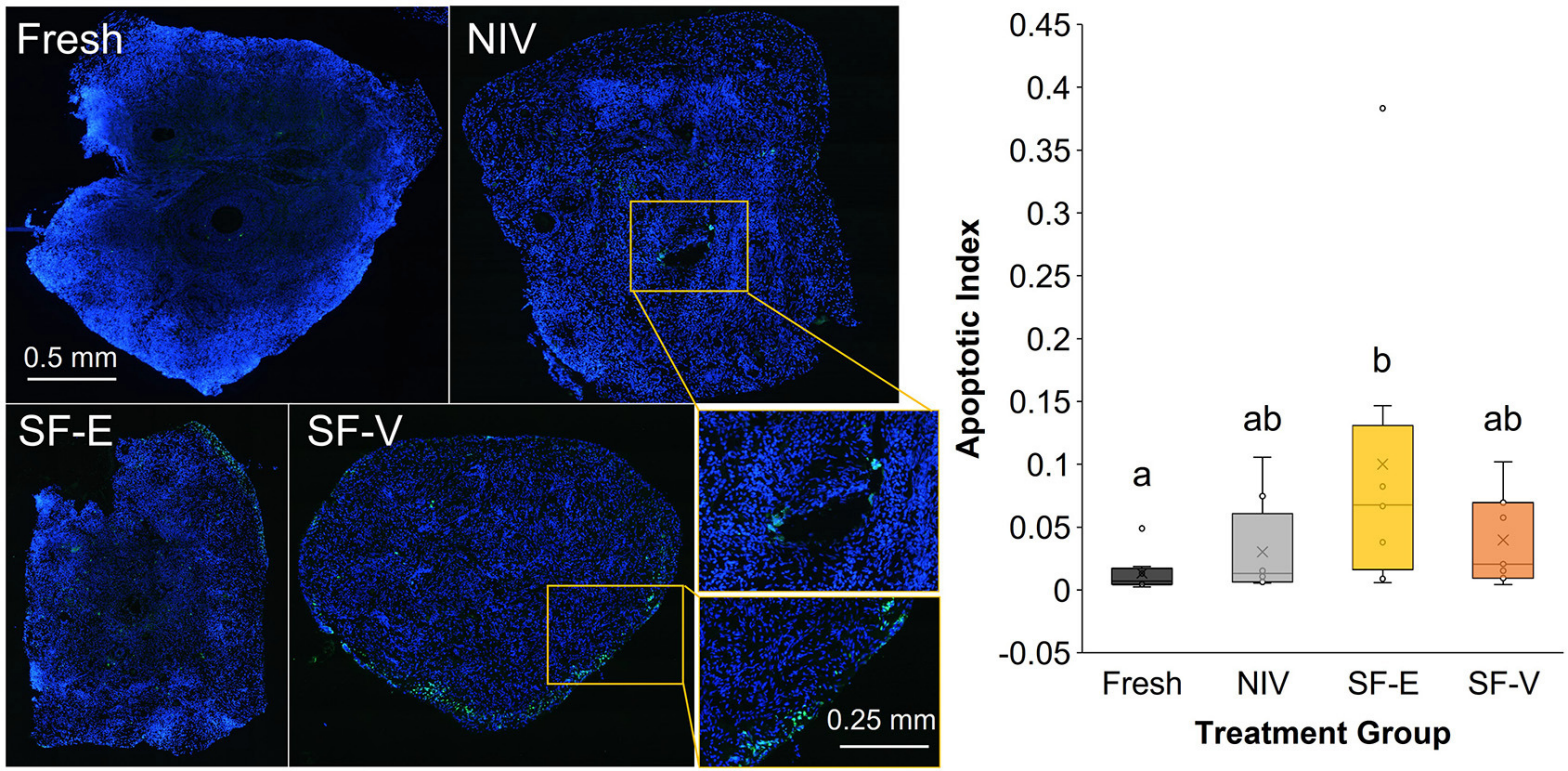
TUNEL stain of fresh and cryopreserved [*via* needle immersed vitrification (NIV), or slow freezing with either 7.5% DMSO and EG (SF-E), or 15% DMSO and EG with 0.5 M sucrose (SF-V)] African painted dog ovarian tissues (*n* = 8), with representative images displaying TUNEL positive cells in green, counterstained with blue DAPI (left) and relative ratio of fluorescence, or Apoptotic Index (right). Box-and-whisker plot displays median, quartile, and data range for follicle densities, with circles representing individuals, with letters indicating statistically significant differences among treatment groups following linear mixed model and *post hoc* Tukey HSD test (*p* < 0.05).

IHC analyses revealed the SF-E tissues had significantly higher apoptotic indices when compared to Fresh tissue (Figure 6), but was not statistically different from NIV or SF-V. Subjectively, apoptosis appeared to be centered around the perforation site of the needle in the NIV tissues, whereas in the SF tissues, apoptosis was primarily noted at the periphery of tissue sections.

## Discussion

In this study, we present the first, to our knowledge, characterization of ovarian follicle morphometrics in the African painted dog, and evaluation of cryopreservation techniques for ovarian tissue. While the sample size (*n* = 11 individuals) was limited due to the opportunistic nature of ovary collection in this endangered species, we identified several key takeaway messages. First, ovarian follicle morphometrics were similar in the African painted dog compared to the domestic dog for late preantral and larger stages of follicles, but larger than what has been described in the dog for early stages of folliculogenesis (13, 24). Second, needle immersion vitrification maintained higher morphologically normal follicle density compared to the slow freezing protocols applied, though all cryopreservation groups experienced increased apoptosis and stromal cell damage. Finally, we did not observe significant changes in expression of select genes associated with oxidative stress and apoptosis among treatment groups.

Our observation that the needle immersed vitrification protocol, if not the slow freezing techniques, maintained follicle density similar to fresh tissue controls was promising. To date, there have been a wide variety of ovarian vitrification and slow freezing protocols applied to large mammalian species; however, methods have varied widely in cryoprotective agents used, their concentrations, and cryoprotectant exposure times, etc. (25), making direct comparisons challenging. For human tissues, vitrification has also been demonstrated to best support primordial follicle populations (26), with the majority of studies utilizing a combination cryoprotectant approach including EG as one of the cryoprotectants (27). This approach is popular due to the evidence that EG is highly cell-permeable (28), minimally toxic (29), and that use of cryoprotectant combinations often allow for less cytotoxicity while reaching the high overall concentrations needed to achieve vitrification (30). There have been only a select few studies in canid species specifically comparing ovarian tissue cryopreservation methods, however. Jivago et al. compared solid surface vitrification (final vitrification in ~15% DMSO, 6% acetamide, and 23% 1,3-propanediol) to slow freezing (~12% DMSO and 11 mM sucrose) for preservation of canine ovarian tissue and found a significantly lower percentage of morphologically normal preantral follicles in vitrified tissue compared with fresh or SF tissue, although an ultrastructural analysis revealed vitrified tissues retained a similar structure to fresh controls (13). Conversely, NIV of domestic dog ovarian tissues with 15% EG, 7.5% DMSO, 0.5 M sucrose and 2.5% non-permeating polymer polyvinylpyrrolidone better supported follicle morphology and developmental competence compared with slow freezing (10% DMSO and 0.1 M sucrose) and vitrification with different combinations of the cryoprotectants (31). Along with the findings of the current study, this suggests that the combined permeating and non-permeating cryoprotectants and ultrarapid freezing techniques are minimally detrimental to the preservation of the canid follicular reserve.

Despite the advantage in maintaining follicular density post-warming, the observation of stromal cell loss and disorganization following all cryopreservation protocols indicates that the utilized NIV methodology requires additional optimization for the African painted dog. In human and murine OTC, less stromal damage has been observed using an NIV protocol compared with slow freezing (10), and in domestic canine ovarian tissue, solid state vitrified tissues had a higher density of normal stromal cells than slow-frozen samples (13). However, the absence of consistent protocols to quantify damage and disorganization in the stromal compartment has challenged our ability to compare across treatment groups, leading us to apply the semi-quantitative assessment described here. Moreover, as the ultimate goal of this work is to utilize the vitrified tissues either *via* grafting/transplantation and/or *in vitro* culture, maintenance of the stromal compartment and its functionality in supporting folliculogenesis will be key to obtain developmentally competent oocytes through either technique.

Similarly, all cryopreserved samples in this study exhibited increased apoptotic indices compared with Fresh controls. This was anticipated, as the toxicity of CPAs alone (32), along with the cell stress associated with cryopreservation and warming, can all contribute to apoptosis. For the SF groups, apoptosis was focused at the periphery of tissues, which may be due to the formation of extracellular ice crystals (33), or the prolonged exposure to CPAs during the slow-cooling process. For NIV, the potential mechanical damage from needle placement (suggested by the apoptotic signal observed around the needle puncture site in some tissues), indicate that alternative methods of vitrification (e.g., solid state) should also be given further consideration. Surprisingly, the SF-V group, which represented the higher CPA concentrations of the vitrification treatment combined with slow freezing exposure times, did not display additional elevations in apoptotic index expected. Rather, the SF-E treated tissues in our study had the highest average apoptotic index when assessed by TUNEL assay. Beyond a higher concentration of the two primary CPAs, DMSO and EG, the primary difference between the treatments was the addition of sucrose to SF-V. As a non-permeating cryoprotectant, sucrose both reduces intracellular water content and adds to solution viscosity, potentially acting to decrease both intracellular and extracellular ice formation. While further exploration is needed, these data suggest that the presence of sucrose may also be beneficial in slow freezing OTC for this species, which has also been observed in slow freezing work in the domestic cat (34).

We also noted a low apoptotic index in fresh tissue overall. The few apoptotic cells observed in these Fresh treatments were typically either associated with the edge of the tissue (likely damage from processing/dissection), or within the granulosa layers of antral follicles, which may be attributed to normal functions in this stage follicle (35). This result indicates that ovary transportation and handling did not overtly impact cell survival. Previous studies have demonstrated that, for most non-human species, transport at 4°C for up to 24 h results in minimal damage [reviewed (36)]. This is important to evaluate for the African painted dog as well, as the ability to quickly receive and process tissues for gamete banking efforts are often limited by distance between zoological institutions and banking facilities. Moreover, research in the domestic dog has determined normal oocyte maturation rates in ovarian tissues transported under similar conditions as applied in the current study (37). Future work should also determine the developmental capacity of the tissue-encapsulated ovarian follicles and oocytes following transport.

No significant differences in expression were observed for any of the genes evaluated. However, previous work by Bebbere et al. showed that ovine ovarian tissues preserved *via* lyophilization displayed significantly reduced RNA integrity compared with fresh controls (38). Though not statistically significant, the SF treatments in the current study had slightly higher beta actin Ct values, suggesting potential RNA degradation, and potentially contributing to the high variation among individuals observed in the post-thaw gene expression. This would be in contrast to observations in human ovarian tissue cryopreservation, wherein vitrification [utilizing 2.62 M DMSO (~20%), 2.60 M acetamide, 1.31 M polypropylene glycol, and 0.0075 M polyethylene glycol solution in a 4 step equilibration process] maintained RNA integrity, whereas a conventional slow-freezing protocol (with 1.5M DMSO and 0.1 M sucrose) resulted in significant degradation (39). Despite the elevation in apoptotic indices, particularly in SF-E, the lack of significant variation in caspase-3 expression among post-thaw tissues was unexpected. However, this result is similar to work in human ovarian tissue, which has observed either no change (40, 41) or a decrease in *CASP3* expression in vitrified tissues compared with fresh (20). Another study evaluated the activity of caspase 3 in collared peccary (Pecari tajacu) ovarian tissue, and found it to be decreased (42) in vitrified samples. While we did not assess caspase 3 activity in this study, the discrepancy observed between the Apoptag/TUNEL staining and *CASP3* results are in line with work in bovine blastocyst (43) and murine testicular tissue (44) vitrification indicating high proportions of post-warming cell death to be attributed to necrosis or autolytic cell death pathways. Importantly, TUNEL assays do not distinguish between these pathways (45). Understanding of the mechanisms preceding the DNA damage observed will be important in the optimization of vitrification protocols for ovarian tissues moving forward.

We had also anticipated that cryopreservation would alter the expression of genes associated with oxidative stress pathways, including *GPX3, SOD1*, and *SOD2*. All three of these genes have been demonstrated to be elevated in 30% DMSO-exposed murine zygotes, even prior to cryopreservation (21). The lack of significant difference in gene expression may again suggest that the combination of cryoprotectants utilized and cryopreservation techniques did not result in major oxidative stress; however, this is contrary to reports of vitrification of oocytes and ovarian tissue in other species. Vitrified mouse oocytes show an increase in *SOD2* expression relative to fresh ova (46) and vitrified domestic dog oocytes demonstrated significant increases in *SOD1* expression relative to fresh controls (47). These differences may be due to variation in vitrification protocols (exposure time, CPA concentration, etc), sample type (cumulus-oocyte complex vs. ovarian tissue) or may reflect species variability in response to these cryoprotectants.

In sum, this study is the first to analyze and attempt to cryopreserve ovarian cortical tissue in the African painted dog. Needle immersed vitrification was proven to be an effective method to further optimize for the cryopreservation of ovarian tissues, where future work should be aimed at reducing stromal loss and developing *in vitro* culture or applying xenografting to evaluate the post-warming functionality of cryopreserved African painted dog ovarian tissue for our genome preservation efforts.

## Data availability statement

The raw data supporting the conclusions of this article will be made available by the authors, without undue reservation.

## Author contributions

KH, CM, and JN performed the experiments. All authors contributed to study design and manuscript preparation. All authors contributed to the article and approved the submitted version.

## References

[1] Woodroffe R Sillero-Zubiri C. Lycaon pictus (amended version of 2012 assessment). The IUCN Red List of Threatened Species 2020: e.T12436A166502262. IUCN Red List (2020).

[2] Leonel ECR Lucci CM Amorim CA. Cryopreservation of human ovarian tissue: a review. Transfus Med Hemother. (2019) 46:173–81. 10.1159/000499054 31244585 PMC6558345

[3] Nakano MS Simoes RS Baracat MCP Lobel A Shiroma ME Igami DZ. Live birth rate after ovarian tissue cryopreservation followed by autotransplantation in cancer patients: a systematic review. Endocrinol Metab. (2020) 1:89–94. 10.53260/grem.201024

[4] Sztein J Sweet H Farley J Mobraaten L. Cryopreservation and orthotopic transplantation of mouse ovaries: new approach in gamete banking. Biol Reprod. (1998) 58:1071–4. 10.1095/biolreprod58.4.1071 9546742

[5] Gosden RG Baird D Wade J Webb R. Restoration of fertility to oophorectomized sheep by ovarian autografts stored at-196 C. Hum Reprod. (1994) 9:597–603. 10.1093/oxfordjournals.humrep.a138556 8046009

[6] Vilela JM Leonel EC Gonçalves LP Paiva RE Amaral RS Amorim CA. Function of cryopreserved cat ovarian tissue after autotransplantation. Animals. (2019) 9:1065. 10.3390/ani9121065 31810266 PMC6941094

[7] Gunasena K Lakey J Villines P Bush M Raath C Critser E. Antral follicles develop in xenografted cryopreserved African elephant (*Loxodonta africana*) ovarian tissue. Anim Reprod Sci. (1998) 53:265–75. 10.1016/S0378-4320(98)00132-8 9835381

[8] Cleary M Shaw J Jenkin G Trounson A. Influence of hormone environment and donor age on cryopreserved common wombat (*Vombatus ursinus*) ovarian tissue xenografted into nude mice. Reprod Fertil Dev. (2005) 16:699–707. 10.1071/RD04054 15740693

[9] Wiedemann C Hribal R Ringleb J Bertelsen M Rasmusen K Andersen C. Preservation of primordial follicles from lions by slow freezing and xenotransplantation of ovarian cortex into an immunodeficient mouse. Reprod Domest Anim. (2012) 47:300–4. 10.1111/rda.12081 23279524

[10] Wang Y Xiao Z Li L Fan W Li S-W. Novel needle immersed vitrification: a practical and convenient method with potential advantages in mouse and human ovarian tissue cryopreservation. Hum Reprod. (2008) 23:2256–65. 10.1093/humrep/den255 18614614

[11] Xiao Z Li SW Zhang Y-Y Wang Y Li L-l Fan W. NIV versus dropping vitrification in cryopreservation of human ovarian tissue. CryoLetters. (2014) 35:226–31. 24997840

[12] Andrae C Oliveira E Ferraz M Nagashima J. Cryopreservation of grey wolf (*Canis lupus*) testicular tissue. Cryobiology. (2021). 10.1016/j.cryobiol.2021.01.010 33482146

[13] Jivago J Paulini F Silva R Araujo M Marinho A Lucci C. Cryopreservation and characterization of canine preantral follicles. Cryobiology. (2018) 81:34–42. 10.1016/j.cryobiol.2018.02.013 29481782

[14] Marin L Bedoschi G Kawahara T Oktay KH. History, evolution and current state of ovarian tissue auto-transplantation with cryopreserved tissue: a successful translational research journey from 1999 to 2020. Reprod Sci. (2020) 27:955–62. 10.1007/s43032-019-00066-9 32046442 PMC7148200

[15] Pukazhenthi BS Nagashima J Travis AJ Costa GM Escobar EN Franca LR. Slow freezing, but not vitrification supports complete spermatogenesis in cryopreserved, neonatal sheep testicular xenografts. PLoS ONE. (2015) 10:e0123957. 10.1371/journal.pone.0123957 25923660 PMC4414479

[16] Mouttham L Comizzoli P. Presence of sucrose in the vitrification solution and exposure for longer periods of time improve post-warming follicle integrity in cat ovarian tissues. Reprod Domest Anim. (2017) 52:224–9. 10.1111/rda.12847 27757998

[17] Songsasen N Wildt DE. Oocyte biology and challenges in developing in vitro maturation systems in the domestic dog. Anim Reprod Sci. (2007) 98:2–22. 10.1016/j.anireprosci.2006.10.004 17097840 PMC1868673

[18] Higgitt RL. Characterization of *Mycobacterium bovis* specific immune responses in African wild dogs (*Lycaon pictus*). M. Sc. Thesis Stellenbosch University, South Africa (2018).

[19] Youle RJ Strasser A. The BCL-2 protein family: opposing activities that mediate cell death. Nat Rev Mol Cell Biol. (2008) 9:47–59. 10.1038/nrm2308 18097445

[20] Dalman A Farahani NSDG Totonchi M Pirjani R Ebrahimi B Valojerdi MR. Slow freezing versus vitrification technique for human ovarian tissue cryopreservation: an evaluation of histological changes, WNT signaling pathway and apoptotic genes expression. Cryobiology. (2017) 79:29–36. 10.1016/j.cryobiol.2017.09.007 28987775

[21] Kang M-H Das J Gurunathan S Park H-W Song H Park C. The cytotoxic effects of dimethyl sulfoxide in mouse preimplantation embryos: a mechanistic study. Theranostics. (2017) 7:4735. 10.7150/thno.21662 29187900 PMC5706096

[22] Pfaffl MW. A new mathematical model for relative quantification in real-time RT-PCR. Nucleic Acids Res. (2001) 29:e45. 10.1093/nar/29.9.e45 11328886 PMC55695

[23] Bates D Mächler M Bolker B Walker S. Fitting linear mixed-effects models using lme4. arXiv [preprint] arXiv:14065823. 2014. 10.18637/jss.v067.i01

[24] Songsasen N Fickes A Pukazhenthi BS Wildt DE. Follicular morphology, oocyte diameter and localisation of fibroblast growth factors in the domestic dog ovary. Reprod Domest Anim. (2009) 44:65–70. 10.1111/j.1439-0531.2009.01424.x 19754538 PMC3257177

[25] Santos R Amorim C Cecconi S Fassbender M Imhof M Lornage J. Cryopreservation of ovarian tissue: an emerging technology for female germline preservation of endangered species and breeds. Anim Reprod Sci. (2010) 122:151–63. 10.1016/j.anireprosci.2010.08.010 20832203

[26] Shi Q Xie Y Wang Y Li S. Vitrification versus slow freezing for human ovarian tissue cryopreservation: a systematic review and meta-analysis. Sci Rep. (2017) 7:1–9. 10.1038/s41598-017-09005-7 28819292 PMC5561141

[27] El Cury-Silva T Nunes ME Casalechi M Comim FV Rodrigues JK Reis FM. Cryoprotectant agents for ovarian tissue vitrification: systematic review. Cryobiology. (2021). 10.1016/j.cryobiol.2021.08.001 34370991

[28] Newton H Fisher J Arnold J Pegg D Faddy M Gosden R. Permeation of human ovarian tissue with cryoprotective agents in preparation for cryopreservation. Hum Reprod. (1998) 13:376–80. 10.1093/humrep/13.2.376 9557842

[29] Gastal G Alves B Alves K Paiva S de Tarso S Ishak G. Effects of cryoprotectant agents on equine ovarian biopsy fragments in preparation for cryopreservation. J Equine Vet Sci. (2017) 53:86–93. 10.1016/j.jevs.2016.03.014

[30] Yong KW Laouar L Elliott JAW Jomha NM. Review of non-permeating cryoprotectants as supplements for vitrification of mammalian tissues. Cryobiology. (2020) 96:1–11. 10.1016/j.cryobiol.2020.08.012 32910946

[31] Fujihara M Kaneko T Inoue-Murayama M. Vitrification of canine ovarian tissues with polyvinylpyrrolidone preserves the survival and developmental capacity of primordial follicles. Sci Rep. (2019) 9:1–11. 10.1038/s41598-019-40711-6 30850725 PMC6408471

[32] Rajaei F Karja N Agung B Wongsrikeao P Taniguchi M Murakami M. Analysis of DNA fragmentation of porcine embryos exposed to cryoprotectants. Reprod Domest Anim. (2005) 40:429–32. 10.1111/j.1439-0531.2005.00585.x 16149947

[33] Courbiere B Odagescu V Baudot A Massardier J Mazoyer C Salle B. Cryopreservation of the ovary by vitrification as an alternative to slow-cooling protocols. Fertil Steril. (2006) 86(4Supplement):1243–51. 10.1016/j.fertnstert.2006.05.019 16978623

[34] Tanpradit N Comizzoli P Srisuwatanasagul S Chatdarong K. Positive impact of sucrose supplementation during slow freezing of cat ovarian tissues on cellular viability, follicle morphology, and DNA integrity. Theriogenology. (2015) 83:1553–61. 10.1016/j.theriogenology.2015.01.035 25747194

[35] Regan SL Knight PG Yovich JL Leung Y Arfuso F Dharmarajan A. Granulosa cell apoptosis in the ovarian follicle—a changing view. Front Endocrinol. (2018) 9:61. 10.3389/fendo.2018.00061 29551992 PMC5840209

[36] Vilela JdMV Dolmans M-M Amorim CA. Ovarian tissue transportation: a systematic review. Reprod Biomed. (2021) 42:351–65. 10.1016/j.rbmo.2020.11.001 33288476

[37] Taş M Evecen M Özdaş ÖB Cirit Ü Demir K Birler S. Effect of transport and storage temperature of ovaries on in vitro maturation of bitch oocytes. Anim Reprod Sci. (2006) 96:30–4. 10.1016/j.anireprosci.2005.11.001 16343825

[38] Bebbere D Arav A Nieddu SM Burrai GP Succu S Patrizio P. Molecular and histological evaluation of sheep ovarian tissue subjected to lyophilization. Animals. (2021) 11:3407. 10.3390/ani11123407 34944182 PMC8697944

[39] Isachenko V Lapidus I Isachenko E Krivokharchenko A Kreienberg R Woriedh M. Human ovarian tissue vitrification versus conventional freezing: morphological, endocrinological, and molecular biological evaluation. Reproduction. (2009) 138:319–27. 10.1530/REP-09-0039 19439559

[40] Abdollahi M Salehnia M Salehpour S Ghorbanmehr N. Human ovarian tissue vitrification/warming has minor effect on the expression of apoptosis-related genes. Iran Biomed J. (2013) 17:179–86. 10.6091/ibj.1243.2013 23999713 PMC3882920

[41] Wiweko B Soebijanto S Boediono A Mansyur M Siregar NC Suryandari DA. Survival of isolated human preantral follicles after vitrification: analyses of morphology and Fas ligand and caspase-3 mRNA expression. Clin Exp Reprod Med. (2019) 46:152. 10.5653/cerm.2019.00143 31813207 PMC6919210

[42] Campos LB da Silva AM Praxedes ECG Bezerra LGP Lins TLBG Menezes VG. Vitrification of collared peccary ovarian tissue using open or closed systems and different intracellular cryoprotectants. Cryobiology. (2019) 91:77–83. 10.1016/j.cryobiol.2019.10.193 31639331

[43] Najafzadeh V Bojsen-Møller Secher J Pihl M Ærenlund A Jørgensen N Jensen KK. Vitrification yields higher cryo-survival rate than slow freezing in biopsied bovine in vitro produced blastocysts. Theriogenology. (2021) 171:44–54. 10.1016/j.theriogenology.2021.04.020 34023618

[44] Hajiaghalou S Ebrahimi B Shahverdi A Sharbatoghli M Boroujeni NB. Comparison of apoptosis pathway following the use of two protocols for vitrification of immature mouse testicular tissue. Theriogenology. (2016) 86:2073–82. 10.1016/j.theriogenology.2016.06.027 27492762

[45] Grasl-Kraupp B Ruttkay-Nedecky B Koudelka H Bukowska K Bursch W Schulte-Hermann R. In situ detection of fragmented DNA (TUNEL assay) fails to discriminate among apoptosis, necrosis, and autolytic cell death: a cautionary note. Hepatology. (1995) 21:1465–8. 10.1002/hep.1840210534 7737654

[46] Amidi F Khodabandeh Z Mogahi MHN. Comparison of the effects of vitrification on gene expression of mature mouse oocytes using cryotop and open pulled straw. Int J Fertil Steril. (2018) 12:61. 10.22074/ijfs.2018.5112 29334209 PMC5767935

[47] Park J-H Kim S-K. Effect of vitrification on in vitro maturation and development and gene expression in canine oocytes. Reprod Dev Biol. (2011) 35:131–6.20565987

